# Evaluation of critical congenital heart disease from 2018 to 2020 in Turkey: a retrospective cohort study

**DOI:** 10.1186/s12884-023-06193-1

**Published:** 2023-12-16

**Authors:** Nilgün Çaylan, Sıddika Songül Yalçın, Başak Tezel, Oben Üner, Şirin Aydin, Fatih Kara

**Affiliations:** 1 Child and Adolescent Health Department, Ministry of Health, General Directorate of Public Health, Ankara, Turkey; 2 Department of Social Pediatrics, Hacettepe University Institute of Child Health, Ankara, Turkey; 3 Department of Public Health, Selçuk University Faculty of Medicine, Konya, Turkey

**Keywords:** Critical congenital Heart Disease (CCHD), Birth cohort, Prevalence, Newborn screening, Case fatality, Survival

## Abstract

**Background:**

The aim of this study is to examine the features of critical congenital heart disease (CCHD).

**Methods:**

The study was planned as a retrospective cohort study. Data for the study were obtained through national data collection systems and 2018–2020 CCHD cohort was established. In this study, we divided the patients into two groups: Group 1 included seven primary target diseases of the newborn CCHD screening program and Group 2 included secondary target diseases.

**Results:**

There were 9884 CCHD cases, with a prevalence of 27.8 per 10,000 live births. Of the cases 44.4% were in Group 1 (12.3 per 10,000) and 54.8% were in Group 2 (15.2 per 10,000). Of all cases 55.5% were male and the female/male ratio was 1/1.2. While 21.8% of the cases were premature, 23.0% were babies with low birth weight (LBW), 4.8% were born from multiple pregnancies. The highest prevalence of CCHD was found in LBW (84.8 per 10,000), premature infants (57.8 per 10,000) (p < 0.001). The fatality rate in the cohort was 16.6% in the neonatal period, 31.6% in the first year of life respectively. The mean estimated survival time in the birth cohort was 40.0 months (95% CI: 39.5–40.6). The mean survival time for Group 1 diseases was 33.4 months (95% CI: 32.5–34.2), while it was 45.4 months (95% CI: 44.7–46.0) for Group 2 diseases (p < 0.001). Preterm birth, LBW, maternal age and region were evaluated as factors associated with mortality risk.

**Conclusion:**

This study showed that CCHDs are common in Turkey and mortality rates are high. There are regional differences in CCHD both prevalence and survival. Improving prenatal diagnosis rates and expanding neonatal CCHD screening are of key importance.

## Introduction

Congenital heart disease (CHD) is malformation of the heart or great vessels that occur during intrauterine development and is the most common group of congenital malformations. They occur in approximately 8–12 of every 1000 live births [1–6]. In a study conducted in the Central Anatolian Region of Turkey, an increase from 6.35 per 1000 live births in 1995 to 9.65 in 2002 was reported (total 7.77) [6].

Critical congenital heart disease (CCHD) is defined as cardiac lesions requiring intervention and/or surgical treatment within the first year of life. It is estimated that approximately 20–25% of all CHDs are in this category [7, 8]. CCHD is associated with high mortality, lifelong morbidity in some types of the disease and high treatment costs [9–11]. The prognosis of CCHD has improved significantly in recent years, although there are significant differences between countries and regions. The improvements in cardiac catheterization in newborns, the developments in surgical and anesthesia techniques, and the increase in care standards in intensive care units have contributed significantly to this improvement [12, 13]. Another important factor that can reduce morbidity and mortality in infants with CCHD is early diagnosis [14–17]. Prenatal diagnosis and postnatal newborn screening with pulse oximetry are two early detection strategies for CCHD in addition to postnatal physical examination [18]. Particularly, prenatal diagnosis of ductus-dependent lesions enables better disease management [19]. Neonatal screening with pulse oximetry is also potentially life-saving in postnatal asymptomatic infants with CCHD who cannot be diagnosed prenatally [14, 20–24].

Recently, the issue of CCHD and its screening is on the agenda in Turkey, and many hospital-based studies have been conducted [25–35]. In addition, newborn pulse oximetry screening for CCHD has been recommended by the Ministry of Health (MoH), but newborn screening test is not mandatory as in many countries [23, 36, 37]. However, the epidemiology of CCHD is a subject that has not been studied sufficiently yet. The aim of this study is to determine the prevalence, case fatality rate, survival-related conditions of CCHD and ultimately, to provide evidence for future preventive strategies.

## Methods

### Data sources

The study was planned as a retrospective cohort study. Data for the study were obtained through the following national data collection systems:

#### National health data system (e-Nabız)

e-Nabız is a personal health data recording and monitoring system coordinated by the MoH, which enables citizens and health workers to securely access health data collected from health institutions. In addition, this health information infrastructure enables the processing of the collected data [38].

#### Death notification system (DNS)

DNS is a system that was implemented in 2013 which allows all deaths to be recorded and monitored on this system. All babies born alive without any limitation of gestational age and birth weight and who die before completing 365 days are recorded in DNS as “infant death”. After all infant deaths are registered in the system, they are examined in detail by the “Provincial Infant Mortality Monitoring Committies”, and after the causes of death are determined, their preventability is studied [39].

#### Birth notification system (BNS)

BNS was created in order to record and monitor all births, and thus, births that take place inside and outside the health institutions and that are declared verbally are recorded in this system [40].

#### Turkish Statistical Institute (TSI) birth statistics

The number of births according to years and some sociodemographic characteristics were obtained from TSI and used in prevalence and rate calculations [41].

### Diagnostic codes

Among the International Statistical Classification of Diseases and Related Health Problems 10th Revision (ICD-10) diagnostic codes, Q20-Q28 codes are used to identify CHDs [42].

There are various definitions of CCHD in different publications in the literature, and the diseases included in the studies also vary according to these definitions [21]. In the Neonatal CCHD Screening Guide published by the MoH, CCHDs were examined under two subheadings according to their probability of detection by newborn screening (a) seven primary target diseases and (b) secondary target diseases [37]. In this study, we divided the patients into two groups according to this classification. Group 1 included generally accepted seven primary target diseases of the newborn screening and Group 2 included secondary target other diseases. We especially wanted to examine group 1 diseases separate for comparability of our study with the published studies. Diseases and codes determined by the study group are summarized in Table 1.

**Table 1:** Distribution prevalence of Critical Congenital Heart Defects types with their ICD-10 codes, Turkey, 2018–2020 (n = 9884)

CCHD type	ICD-10	CCHD, n (%)*	Prevalence of CCHD (1/10,000 live birth)
**Overall**		9884 (100.0)	27.8
* Group 1 diseases*		4392 (44.4)	
Tetralogy of Fallot	Q21.3	1721 (17.4)	4.8
Hypoplastic left heart syndrome	Q23.4	929 (9.4)	2.6
Transposition of great arteries	Q20.3	825 (8.3)	2.3
Total anomalous pulmonary venous return	Q26.2	388 (3.9)	1.1
Pulmonary atresia with intact ventricular septum	Q22.0	200 (2.0)	0.6
Truncus arteriosus	Q20.0	199 (2.0)	0.6
Tricuspid atresia	Q22.4	130 (1.3)	0.4
* Group 2 diseases*		5415 (54.8)	15.2
Aortic arch anomalies **	Q25.1,Q25.2, Q25.4	2098 (21.2)	5.9
Atrioventricular septal defect	Q21.2	2021 (20.4)	5.7
Pulmonary valve stenosis	Q22.1	672 (6.8)	1.9
Single ventricle physiology diseases ***	Q20.1, Q20.2, Q20.4, Q22.6	490 (5.0)	1.4
Ebstein anomaly	Q22.5	134 (1.4)	0.4
* Ungrouped*		77 (0.8)	0.2

* Column percentage

** Aortic arch anomalies: Coarctation of the aorta, interrupted aortic arch. aortic atresia/hypoplasia

*** Single ventricle physiology diseases: double outlet right ventricle, double outlet left ventricle,

double inlet left ventricle. hypoplastic right heart syndrome

ICD: International Classification of Diseases

Ventricular septal defect (VSD) cases were excluded from the study since it would be difficult to identify patients who met the definition of CCHD with the data used. Aortic coarctation, interrupted aortic arch, aortic atresia/hypoplasia cases are grouped under the heading of “aortic arch anomalies”; Diagnoses of double outlet right ventricle, double outlet left ventricle, double inlet left ventricle and hypoplastic right heart syndrome were grouped under the heading “Diseases with single ventricle physiology” [37].

### Birth numbers

The number of live births for prevalence calculations was obtained from TSI [41]. The number of births by gender, maternal age at birth, maternal education, parity, number of fetuses, region, gestational age, birth weight, and delivery type were obtained from TSI and BNS.

### CCHD prevalence

All health institution applications containing the Q20-Q28 ICD-10 codes from the birth to the time of data collection of the Turkish citizens born in 2018–2020 were received from the e-Nabız system. The dataset included the following information: Date of birth, date of death, gender, nationality, province of residence, ICD-10 diagnostic codes, date of diagnosis, name of health institution, province where the health institution is located, intervention/surgery applied.

**Fig. 1:**
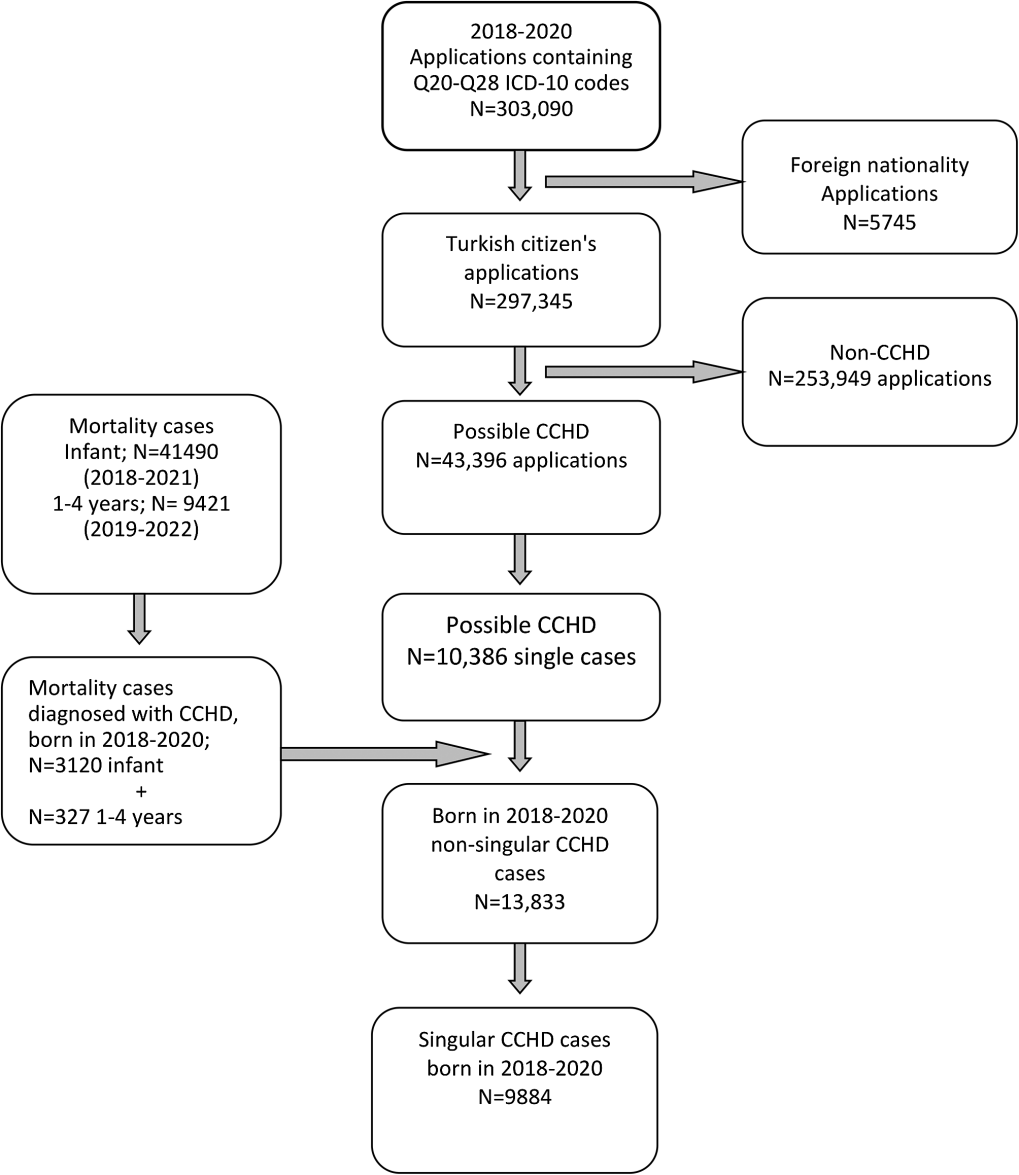
2018–2020 CCHD cohort cases study flow

In the first stage, applications that do not belong to Turkish citizens were eliminated and more than one application of the patients was singularized. Then, “possible CCHD” cases were identified. In the second stage, DNS data for the years 2018–2022 were examined in terms of infant and child deaths (0–4 years) born in 2018–2020 and related to CCHD. This new 0–4 year old CCHD mortality list was then combined with the e-Nabız data obtained in the first stage. The combined list was again deduplicated using citizenship numbers. All cases were analyzed in detail with e-Nabız and DNS information, and cases with a confirmed diagnosis of CCHD were included in the study (Fig. 1). Maternal age, birth weight, gestational age, number of fetuses, mode of delivery data of the cases whose diagnosis was confirmed were obtained from DNS and BNS. The mortality status was updated on December 31, 2022. As a result, the list of Turkish citizens with CCHD diagnosis in 2018–2020 birth cohort was created in a way that all cases would be unique and include up-to-date information.

While prematurity was defined as < 37 gestational week at birth, low birth weight (LBW) was defined as < 2500 g birth weight. Birth weight for gestational age was analyzed in three categories: SGA (small for gestational age, birth weight < 10th percentile), AGA (appropriate for gestational age, birth weight 10-90th percentile), and LGA (large for gestational age, birth weight > 90 percentile) [43]. Mortality cases were categorized by the time of death: early neonatal (0–6 days); late neonatal (7–28 days); post-neonatal period (29–364 days) and ≥ 1 year. Province of residence data was grouped according to the definition of five demographic regions in the Turkey Demographic and Health Survey (TDHS): West, South, Central, North, and East [44].

### Analysis of the data

Data were analyzed using Microsoft Office Excel 2019 and IBM SPSS Statistics for Windows, Version 23.0 statistical software package. Arithmetic mean and standard deviation were used for continuous variables, and frequency and percentage distributions were used for categorical variables. The chi-square test was used when comparing the percentage distribution of categorical data between groups. When significant difference was detected in the 4 × 2, 3 × 2, 2 × 3 variables (p < 0.05), residual analyzes were performed to determine the subgroups that made a difference.

Survival length according to case characteristics in the birth cohort were analyzed by Kaplan Meier analysis, mean and 95% confidence interval (CI) were given, and Log Rank test was used to determine the difference. Relationship between region, gender, gestational age, birth weight according to gestational week, pregnancy type, maternal age and mode of delivery with mortality risk in CCHD cases was analyzed with Cox logistic regression analysis, and a 95% CI was given with adjusted odds ratio (AOR). Significance was accepted as p < 0.05.

## Results

The work flow of the study is given in Fig. 1. Analysis of multiple data systems showed that in the 2018–2020 birth cohort, there were 9884 CCHD cases out of 3,559,603 live births (27.8 per 10,000 live births or 1/360) (Table 1). While 54.8% (n = 5415, 15.2 in 10,000) of CCHD cases were in Group 2, 44.4% (n = 4392, 12.3 in 10,000) were diseases in Group 1. Of the cases 0.8% (n = 77) could not be grouped. Aortic arch anomalies (n = 2098), atrioventricular septal defect (n = 2021) and Tetralogy of Fallot (n = 1721) were the three most common diseases and these constituted 58.8% of all cases (Table 1).

Table 2 shows the distribution of cases with CCHD according to some sociodemographic variables. Of all cases 55.5% (n = 5485) were male and the female/male ratio was 1/1.2. While 21.8% of the cases were premature (n = 2155), 23.0% (n = 2278) were babies with LBW, 4.8% (n = 474) were born from multiple pregnancies. In most of the cases, maternal age at birth was between 20 and 34 years (72.5%) and the majority (64.1%) were born by cesarean section. While most of the cases were in the Western region (n = 3814; 38.6%), the least cases were found in the North (n = 549; 5.6%).

**Table 2:** Critical Congenital Heart Defects’ prevalence by some sociodemographic characteristics, Turkey, 2018–2020

Variables	CCHD, n (%)*	CCHD, prevalence**
n	9884	27.8
**Gender**		
Male	5485 (55.5)	30.0^a^
Female	4399 (44.5)	25.4^b^
**Maternal age at birth**		
< 20 years	404 (4.1)	25.1^a^
20–34 years	7170 (72.5)	25.6^a^
≥ 35 years	2250 (22.8)	39.8^b^
Unknown	60 (0.6)	
**Number of fetuses in pregnancy**		
Singular	9305 (94.1)	27.1^a^
Twin/triplet	474 (4.8)	42.5^b^
Unknown	110 (1.1)	
Gestational age***		
≥ 37 weeks	7620 (77.1)	24.1^a^
< 37 weeks	2155 (21.8)	55.0^b^
Unknown	109 (1.1)	
Birth weight***		
≥ 2500 gram	7497 (75.8)	22.8^a^
< 2500 gram	2278 (23.0)	83.1^b^
Unknown	109 (1.1)	
Mode of delivery***		
Normal	3208 (32.4)	22.7^a^
Cesarean	6332 (64.1)	29.5^b^
Unknown	344 (3.5)	─
**Regions**		
West	3814 (38.6)	29.5^a^
South	1288 (13.0)	28.5^a^
Central	1826 (18.5)	28.2^a^
North	549 (5.6)	30.3^a^
East	2352 (23.8)	24.0^b^
Unknown	55 (0.6)	─

^a.b^ Different letters in the same column are statistically significant (p < 0.001)

*column percentage

**1/10,000 live birth;

***The data for gestational age, birth weight, delivery type was only available in the 2020 Birth Notification System. In order to estimate the number of live births according to gestational age, birth weight and mode of delivery, the 2018–2020 relative percentages of these variables were calculated with the 2020 distributions. Then, CCHD prevalence were estimated with this values. Live birth data for other parameters were present in 2018–2020

### Prevalence of CCHD by sociodemographic characteristics

Table 2 also shows the change in the prevalence of CCHD according to some sociodemographic characteristics. Maternal age (≥ 35 years), number of fetuses in pregnancy, premature birth, LBW, male gender, and cesarean delivery were associated with a higher incidence of CCHD (p < 0.001). The highest incidence of CCHD was found in LBW (84.8 per 10,000 live births), premature (57.8 per 10,000 live births), and twin/triplet (42.5 per 10,000 live births) infants. When the regional differences are examined, the lowest CCHD prevalence was found in the East (24.0 out of 10,000), while the highest was in the North (30.3 out of 10,000) (Table 2).

### Mortality in the CCHD birth cohort

**Fig. 2:**
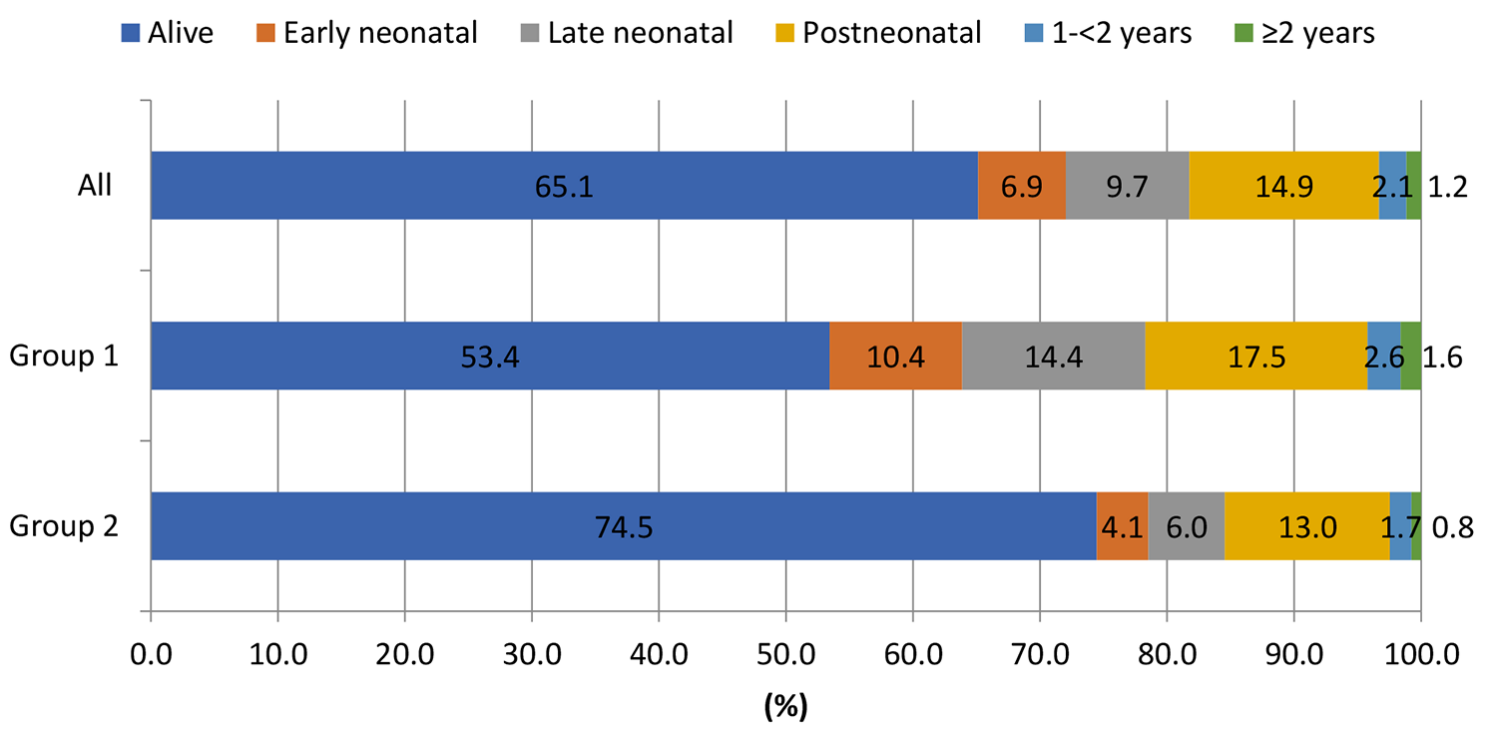
Survival in the CCHD birth cohort and distribution by age at which mortality occured, 2018–2020, Turkey (n = 9884)

The fatality rate in the cohort was 6.9% (682/9884) in the early neonatal period, 16.6% (1643/9884) in the neonatal period, 31.6% (3120/9884) in the first year of life, and 33.7% (3332/9884) in the two years of life. The distribution by age at which mortality occurred is as follows: Early neonatal 6.9%; late neonatal 9.7%; 14.9% in the postneonatal period and 2.1% in the ≥ 1 -<2 years and 1.2% in the ≥ 2years of age group. While mortality was 42.3% in the first year of life in Group 1 patients, it was 23.1% in Group 2 diseases (Fig. 2).

Diseases with the highest mortality rate in the birth cohort are respectively; hypoplastic left heart syndrome (HLHS) (86.9%), truncus arteriosus (59.8%), and pulmonary atresia with intact ventricular septum (59.0%).

### Survival in the CCHD birth cohort

Survival status and survival length by CCHD groups associated with some variables according to CCHD case groups are shown in Table 3.

**Table 3:** Survival status and survival length by the group of Critical Congenital Heart Defects (month)*

CCHD group	Variables	Total n	Alive %	Mean (months) (95% CI)	Log Rank (Mantel-Cox), p
**Total**		9884	65.1	40.0 (39.5–40.6)	
**Group 1**		4392	53.4	33.4 (32.5–34.2)^a^	< 0.001
**Group 2**		5415	74.5	45.4 (44.7–46.0)^b^	
	**Gender**				
Group 1	*Female*	1820	54.6	34.1 (32.8–35.4)	0.095
	*Male*	2572	52.6	32.8 (31.7–33.4)	
Group 2	*Female*	2550	73.9	45.1 (44.1–46.0)	0.370
	*Male*	2865	75.0	45.6 (44.7–46.5)	
	**Gestational age**				
Group 1	*< 37 weeks*	885	39.0	25.0 (23.1–26.8)	< 0.001
	*≥ 37 weeks*	3447	56.5	35.1 (34.2–36.1)	
Group 2	*< 37 weeks*	1254	64.1	39.3 (37.8–40.8)	< 0.001
	*≥ 37 weeks*	4118	77.6	47.1 (46.4–47.9)	
	**Birthweight by gestational age**				
Group 1	*SGA*	1064	44.5	28.5 (26.8–30.2)^a^	< 0.001
	*AGA*	3065	55.7	34.5 (33.6–35.5)^b^	
	*LGA*	203	55.2	32.8 (29.1–36.5^)b^	
Group 2	*SGA*	1256	63.6	39.3 (37.8–40.8)^a^	< 0.001
	*AGA*	3771	77.7	47.1 (46.4–47.9)^b^	
	*LGA*	345	78.3	47.2 (44.8–49.7)^b^	
	**Number of fetuses in pregnancy**				
Group 1	Singular	4123	53.4	33.3 (32.4–34.2)	0.007
	Twin/triplet	209	44.0	28.2 (24.4–31.9)	
Group 2	Singular	5107	74.6	45.5 (44.8–46.1)	0.077
	Twin/triplet	264	70.5	42.4 (39.3–45.5)	
	**Maternal age **				
Group 1	*<20 years*	200	47.0	29.5 (25.6–33.4)^a^	0.017
	*20–34 years*	3293	52.7	31.9 (31.9–33.9)^a^	
	≥ 35 years	864	56.5	35.2 (33.3–37.0)^b^	
Group 2	*< 20 years*	201	71.1	43.4 (39.9–46.9)^a.b^	< 0.001
	*20–34 years*	3812	76.4	46.4 (45.6–47.1)^a^	
	*≥35 years*	1377	69.4	42.7 (41.2–44.0)^b^	
	**Mode of delivery**				
Group 1	*Normal*	1447	52.0	32.6 (31.2–34.1)	0.565
	*Cesarean*	2765	51.7	32.3 (31.2–33.3)	
Group 2	*Normal*	1732	73.3	44.8 (43.7–46.0)	0.611
	*Cesarean*	3524	74.2	45.1 (44.3–46.0)	
	**Region**				
Group 1	*West*	1653	56.1	34.7 (33.3–36.1)^a^	0.001
	*South*	575	54.6	34.1 (31.9–36.4)^ab^	
	*Central*	784	50.3	31.7 (29.7–33.7)^bc^	
	*North*	226	61.9	37.9 (34.4–41.4)^a^	
	*East*	1144	49.0	31.0 (29.4–32.6)^c^	
Group 2	*West*	2154	80.0	48.3 (47.4–49.3)^a^	< 0.001
	*South*	703	72.8	44.3 (42.5–46.1)^b^	
	*Central*	1040	75.1	45.9 (44.4–47.3)^b^	
	*North*	319	74.3	45.1 (42.4–47.8)^b^	
	*East*	1188	64.7	40.0 (38.5–41.6)^c^	

CI: Confidence Interval. *The survival length of the groups were compared with Kaplan Meier analysis and Log Rank (Mantel-Cox) test. SGA: Small for gestational age. AGA: Appropriate for gestational age. LGA: Large for gestational age

The mean survival time in the birth cohort was 40.0 months (95% CI: 39.5–40.6). The mean estimated survival time for Group 1 diseases was 33.4 months (95% CI: 32.5–34.2), while it was 45.4 months (95% CI: 44.7–46.0) for Group 2 diseases (p < 0.001, Table 3). Prematurity and SGA at birth was associated with shorter survival for both Group 1 and Group 2 diseases (p < 0.001). Twin/triplet pregnancy was associated with shorter survival only in Group 1 diseases compared to singleton pregnancy (28.2 months 95% CI: 24.4–31.9 vs 33.3 months 95% CI: 32.4–34.2) (p = 0.007). In Group 1 diseases, maternal age ≥ 35 years was associated with a longer survival than infants born to mothers < 20 and 20–34 years old (35.2; 95%CI: 33.3–37.0) (p = 0.017). Infants of mothers aged 20–34 years in Group 2 diseases had a longer survival compared to mothers aged ≥ 35 years (46.4 months; 95% CI: 45.6–47.1 vs 42.7 months; 95% CI: 41.2–44.0) (p < 0.001). There was no difference in survival times depending on gender and mode of delivery (Table 3).

When the regional changes are examined in terms of survival; for Group 1 diseases, the shortest survival time was found in the East (31.0 months) and the Central region (31.7 months), while the longest survival was found in the North with 37.9 months (p = 0.001). While the longest survival for Group 2 diseases was in the West region (48.3 months), the shortest survival time was found in the East region (40.0 months, p < 0.001) (Table 3).

**Fig. 3:**
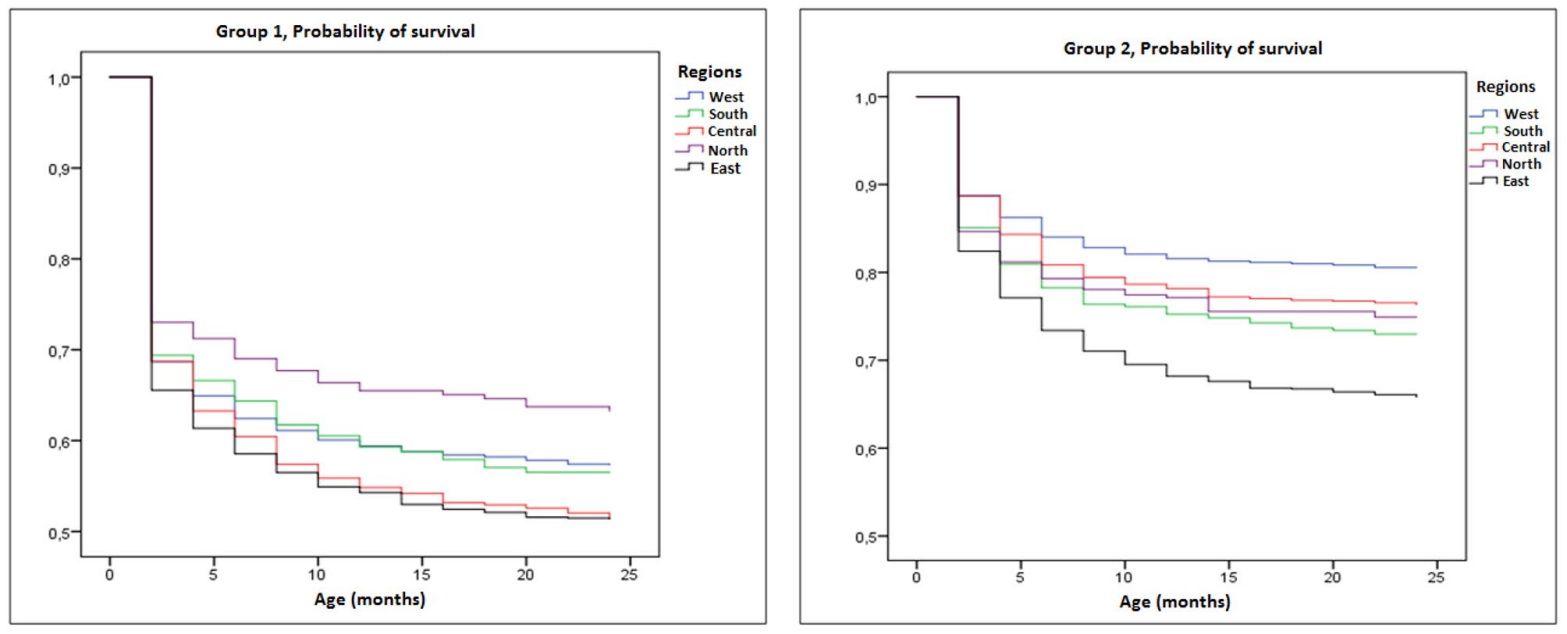
Variation of 24-month survival due to Group 1 and 2 diseases by region

The variation of 24-month survival due to Group 1 and 2 diseases by regions is shown in Fig. 3.

**Fig. 4:**
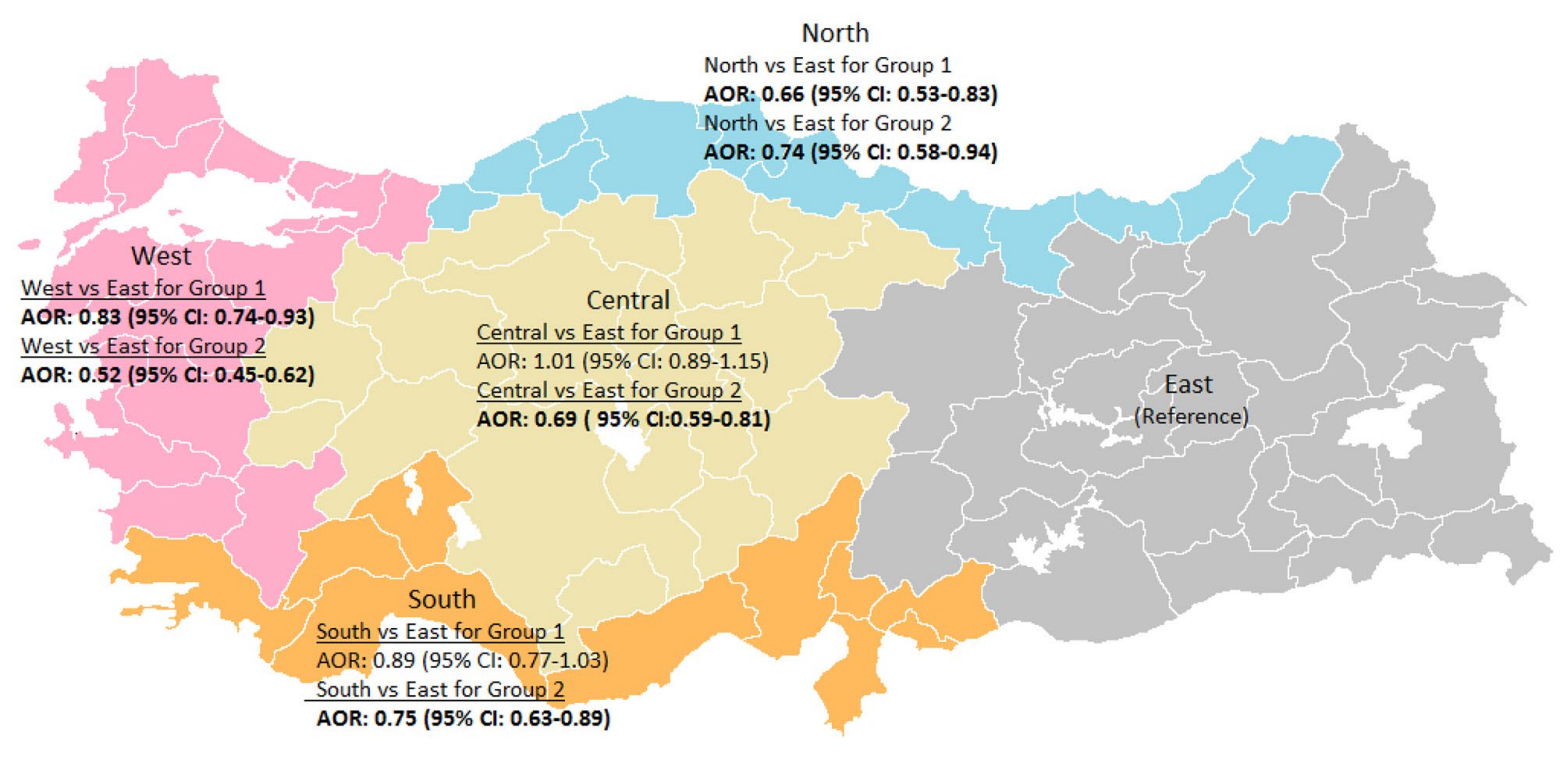
The mortality risks of critical congenital heart disease by region of Turkey

Mortality risk was evaluated by examining region, maternal and infant characteristics together with Cox regression analysis in CCHD groups (Table 4). For Group 1 diseases, compared to the East, living in the West and North regions reduced the risk of mortality by 17% (95% CI: 0.74–0.93) and 34% (95% CI: 0.53–0.83), respectively (p < 0.001, Fig. 4). Prematurity increased the risk of mortality 1.68 fold compared to term delivery (95% CI: 1.52–1.87) (p < 0.001), while being SGA at birth increased 1.35 fold (95% CI: 1.07–1.69) compared to LGA (p = 0.010). Compared with maternal age > 35 years, <20 years maternal age was associated with a 1.33-fold increase in mortality (95% CI: 1.07–1.65; p = 0.010), while maternal 20–34 years age was associated with a 1.18-fold increase (95% CI: 1.05–1.32; p = 0.004) (Table 4).

**Table 4:** Mortality risk in groups of Critical Congenital Heart Defects with multivariable logistic regression

	Group 1 (n = 4190)			Group 2 (n = 5242)		
	AOR	95% CI	p	AOR	95% CI	p
**Region**						
West vs. East	0.83	0.74–0.93	0.001	0.52	0.45–0.60	< 0.001
South vs. East	0.89	0.77–1.03	0.129	0.75	0.63–0.89	0.001
Central vs. East	1.01	0.89–1.15	0.902	0.69	0.59–0.81	< 0.001
North vs. East	0.66	0.53–0.83	< 0.001	0.74	0.58–0.94	0.012
**Gender**						
Female vs. Male	0.92	0.84-1.00	0.055	1.04	0.93–1.15	0.509
**Gestational age**						
< 37 vs. ≥ 37 weeks	1.68	1.52–1.87	< 0.001	1.94	1.72–2.18	< 0.001
**Birth weight by gestational age**						
SGA vs. LGA	1.35	1.07–1.69	0.010	1.85	1.45–2.37	< 0.001
AGA vs. LGA	1.02	0.82–1.26	0.867	1.06	0.84–1.34	0.636
**Number of fetuses in pregnancy**						
Singular vs. Twin/triplet	1.12	0.92–1.37	0.266	1.23	0.97–1.57	0.091
**Maternal age at birth**						
< 20 vs. ≥ 35 years	1.33	1.07–1.65	0.010	0.84	0.63–1.10	0.207
20–34 vs. ≥ 35 years	1.18	1.05–1.32	0.004	0.80	0.72–0.90	< 0.001
**Mode of delivery**						
Normal vs. Cesarean delivery	1.00	0.91–1.10	0.966	1.06	0.94–1.19	0.325

CI: Confidence interval, AOR: Adjusted odds ratio, SGA: Small for gestational age. AGA: Appropriate for gestational age. LGA: Large for gestational age

For Group 2 diseases; living in all regions has been shown to have lower odds of mortality when compared to the East: West 48%, Central 31%, North 26% and South 25% (p < 0.001, p < 0.001, p = 0.012, p < 0.001, respectively, Fig. 4). Compared with term infants, prematurity and SGA at birth compared with LGA increased the probability of mortality 1.94-fold (95% CI: 1.72–2.18; p < 0.001) and 1.85-fold (95% CI: 1. 45-2.37; p < 0.001) respectively. In addition, it was shown that the mortality risk was 20% less in the babies of mothers aged 20–34 years when compared to the babies of mothers aged ≥ 35 years old at birth (95% CI:0.72–0.90; p < 0.001) (Table 4).

Gender, number of fetuses in pregnancy, and mode of delivery were not associated with mortality risk both Group 1 and Group 2 diseases.

## Discussion

In this study using national data, it was shown that the prevalence of CCHD is 27.8 per 10,000 live births. The prevalence of CCHD differs in various countries and regions around the world [19, 22, 45, 46]. In the study, which analyzed data from births between 2010 and 2017 from the Birth Defects Monitoring Network in Beijing, seven CCHDs, the primary target of pulse oximetry screening, were included in the study [46]. In this study, the prevalence of seven diseases in Group 1 was reported as 10.43 out of 10,000, and similarly, the prevalence found for this group in our study was 12.3 out of 10,000. In a study including 11 CCHDs conducted in Nevada, USA in 2016–2019, the prevalence was reported as 18 per 10,000 live births [45]. In addition, while the rate of prenatal diagnosis was 81% in the study, the frequency of being discharged undiagnosed despite pulse oximetry scanning was reported as 4% [45]. In the study in which the results of 15 congenital anomaly monitoring programs from 12 countries in Europe, North and South America and Asia were analyzed together, 18,243 cases of CCHD were reported out of 8,847,081 deliveries. The mean frequency was found to be 19.1 (min:10.1-max:31.0) per 10,000 births. The rate of prenatal diagnosis was at least 50% in one third of the programs, while the lowest rate of prenatal diagnosis was found in Slovakia (13%), 87% in parts of France [19]. In the study, it was also emphasized that the frequency is high in countries where termination of pregnancy (ToP) is not allowed for medical reasons [19]. In a study evaluating the data provided by the National Congenital Anomalies Network in Argentina between 2009 and 2018, the prevalence of CHD was found to be 11.46 (95% CI: 11.02–11.92) per 10,000 births. It has been reported that 43.93% of the cases were diagnosed in the prenatal period [22]. Differences in the results of these studies may be due to time, geography, characteristics of the society in which the research was conducted, as well as methodological differences such as case definition, diseases included, data collection criteria, and study design.

In recent years, although CCHD mortality has decreased gradually, the mortality of this group of diseases is still high [8, 11, 19, 26, 27, 47]. Our study showed that while the mortality rate in the birth cohort was 16.6% in the neonatal period, it reached 31.6% and 33.7% at the end of the first and second years, respectively. The survival rate in Group 1 diseases was, as expected, significantly lower than in Group 2 diseases. In the study in which the results of fifteen congenital anomaly monitoring programs in Europe, North and South America and Asia were analyzed, it was reported that there were significant differences between countries in terms of mortality in the first month of life [19]. The highest neonatal mortality rate in the study was in Argentina (25.5%) and Malta (24.1%). It has been suggested that the fact that the ToP is not allowed in these countries and low prenatal CCHD diagnosis rate may be related to the high mortality rates [19]. In countries and regions where ToP is allowed, CCHD related neonatal mortality was relatively lower and ranged from 4.0 to 11.1%. In Turkey prenatal diagnosis rates are still not optimal [25, 26]. In addition, although there is no legal obstacle to ToP for medical reasons, it is common that families do not accept ToP even in situations incompatible with life [48, 49]. As an important development in recent years, newborn pulse oximetry screening for CCHD is recommended by the MoH and the screening flow chart was added to the Infant Child Adolescent Follow-up Protocols in 2018 [36]. The national guideline that sets the standards for neonatal screening was published in 2021 [37]. However, newborn screening test is not compulsory as in many countries of the World, the program is still implemented as multicenter studies and pilot programs [23].

In Norway between 2014 and 2016, the mortality rate was 10% in 2359 live-born babies with severe CHD, 58% of them died before surgery and 81% of preoperative deaths were during palliative care. Comorbidity and univentricular CHDs have been reported to be common among these infants [50]. Similarly, the disease with the highest mortality in our study was HLHS with a mortality rate of 81.9%. In our study, prematurity, SGA at birth, multiple pregnancy, maternal age at birth, CCHD group are the variables associated with survival in line with the literature [11, 26, 27, 51]. In a study conducted in Brazil, prematurity, LBW, multiple pregnancy, comorbidity/congenital anomaly were associated with increased mortality in CCHD [11]. In another study from Turkey evaluating 105 patients with CCHD in a tertiary neonatal intensive care unit between 2010 and 2012, the mortality rate was reported as 35.2%. In the study, it was concluded that mortality was high in cases with CCHD requiring intervention, and that low gestational week and high interventional risk score (RACHS-1) increased the risk of mortality [27]. In another center in Turkey, in cases requiring intervention in the neonatal period in 2017–2018, overall mortality was reported as 27% and intervention mortality as 22%. LBW, prematurity, need for resuscitation, need for inotropic support and additional organ anomaly were associated with mortality [26].

Regional differences in CCHD prevalence and survival were among the significant outcomes of our study. The prevalence of CCHD was found to be significantly lower in the Eastern region compared to other regions. This may be due to the deficiencies in the diagnosis and reporting of the disease in the Eastern region, as well as due to different factors (such as environmental pollution, climate changes, consanguineous marriages, genetic predisposition) affecting the prevalence of the disease. In terms of survival, the East and Central regions had the least survival in Group 1 diseases, while the East region was the most disadvantaged region in Group 2 diseases. The results of the study indicate that it would be useful to examine regional differences in terms of prevalence and survival in future studies.

The study has several limitations. The independent variables examined were limited to the available variables in the database. For this reason, some independent variables that may be associated with the clinical course, mortality or survival of CCHD were not present in the study (prenatal diagnosis, postnatal screening, time of diagnosis, frequency of late diagnosis, referral status, transport conditions, comorbidity, other accompanying anomalies etc.). Diagnoses from the database are directly related to the knowledge and awareness of health professionals about the use of disease-specific ICD-10 codes. On the other hand, there are some strengths of this study. Our study includes 3-year national birth cohort data and has national representation. Although there is no system for the monitoring of congenital anomalies in Turkey, cohort data could be obtained through other systems that regularly collect data.

In conclusion, this study showed that CCHDs are common in Turkey and mortality rates are high compared to previous studies. Regional differences in CCHD prevalence and survival are striking. In terms of prevalence, it was determined that the East Region had the least frequency, while the probability of mortality was found to be higher in this region compared to other regions. Prematurity, SGA at birth, multiple pregnancy, maternal age at birth, CCHD group are found the variables associated with the survival.

This study has provided to reveal the problem. However, further studies that investigate the situations related to mortality and survival in detail and provide tailored solutions to reduce mortality rates are needed. Expansion of prenatal diagnosis and newborn CHD screening, the effectiveness of which has been proven in previous studies, should be a priority in terms of reducing both mortality and morbidity. It is also necessary to establish a congenital anomaly surveillance system in order to follow up congenital anomalies and measure the effectiveness of preventive interventions.

## Data Availability

Data can be requested from Ministry of Health (contact details: hsgm.ces@saglik.gov.tr).
